# Exploring self-reported visual function and vision-related anxiety in patients with *RPGR*-associated retinal degeneration

**DOI:** 10.1038/s41598-024-66170-2

**Published:** 2024-07-02

**Authors:** Nuno Gouveia, Oluji Chukwunalu, Carolina Oliveira, C. Henrique Alves, Rufino Silva, Joaquim Murta, João Pedro Marques

**Affiliations:** 1 Department of Ophthalmology, Hospitais da Universidade de Coimbra, ULS de Coimbra, Portugal; 2 Faculty of Medicine, University of Coimbra (FMUC), Coimbra, Portugal; 3 Clinical Academic Center of Coimbra (CACC), Coimbra, Portugal; 4 Coimbra Institute for Clinical and Biomedical Research (iCBR), Faculty of Medicine, University of Coimbra (FMUC), Coimbra, Portugal; 5 Faculty of Psychology and Education Sciences, University of Coimbra (FPCEUC), Coimbra, Portugal; 6 Center for Innovative Biomedicine and Biotechnology (CIBB), University of Coimbra, Coimbra, Portugal; 7 Association for Innovation and Biomedical Research on Light and Image (AIBILI), Coimbra, Portugal

**Keywords:** Patient-reported outcomes, Retinitis pigmentosa, RPGR, Anxiety, Disability, Eye manifestations, Eye diseases, Psychology, Quality of life

## Abstract

Variants in the retinitis pigmentosa GTPase regulator (*RPGR*) gene are responsible for the majority of X-linked retinitis pigmentosa cases, which not only affects male patients but also some heterozygous females. Vision-related disability and anxiety of patients with *RPGR*-associated retinal degeneration have never been explored before. This study aimed to evaluate self-reported visual function and vision-related anxiety in a Portuguese cohort of male and female patients with *RPGR*-associated retinal degeneration using two validated patient-reported outcome measures. Cross-sectional data of thirty-two genetically-tested patients was examined, including scores of the Michigan retinal degeneration questionnaire (MRDQ) and Michigan vision-related anxiety questionnaire. Patients were classified according to retinal phenotypes in males (M), females with male phenotype (FM), and females with radial or focal pattern. Both M and FM revealed higher rod-function and cone-function anxiety scores (*p* < 0.017). Most MRDQ disability scores were higher in M and FM (*p* < 0.004). Overall, positive correlations (*p* < 0.004) were found between every MRDQ domain and both anxiety scores. In *RPGR*-associated retinal degeneration, males and females with male phenotype show similar levels of increased vision-related anxiety and disability. Every MRDQ visual function domain showed a strong correlation with anxiety scores.

## Introduction

Retinitis pigmentosa (RP) encompasses a diverse group of inherited retinal disorders characterized initially by night blindness and visual field constriction due to the loss of rod photoreceptors^1–3^. As the disease progresses, cone photoreceptors deteriorate, typically resulting in the loss of color vision, contrast sensitivity, and central vision^1–3^. Retinitis pigmentosa stands as the most prevalent inherited retinal disease (IRD) globally, affecting an estimated 1 in 4000 individuals, and X-linked retinitis pigmentosa (XLRP) accounts for 5–15% of all cases^2,4^. It is one of the most severe forms of RP in males, with symptoms appearing in childhood and progressing rapidly to severe vision impairment by the fourth decade of life^4^. Clinically significant variants in the retinitis pigmentosa GTPase regulator (*RPGR*) gene are responsible for 70–80% of XLRP cases^1^. Despite the X-linked inheritance, heterozygous females of *RPGR* variants may display varying degrees of severity, often with significant inter-eye asymmetry^5^. This phenotypic heterogeneity is thought to stem from differences in the X chromosome inactivation ratio (X lyonization)^4,6^. Fundus autofluorescence (FAF) is a valuable imaging technique for identifying retinal mosaicism and classifying the disease into four patterns of increasing severity: normal, radial, focal and male pattern^4^. Additionally, visual function correlates with retinal phenotype on FAF, with patients showing normal, focal, or radial patterns more likely to retain good visual acuity^5^.

The approval of Voretigene neparvovec for *RPE65*-associated retinal degeneration has promoted research into treatments for other IRD^7^. Several clinical trials are underway, aiming to deliver a healthy *RPGR* gene copy using adeno-associated viral vectors^8–10^. Given the severity and prevalence of this condition, effective treatments are urgently needed to prevent, halt, or reverse disease progression^7,9^. Female carriers of *RPGR* variants, who may exhibit a severe male-like phenotype, could benefit from gene replacement therapies and should be equally considered for future therapeutic approaches, as has already happened in the phase 3 LUMEOS study (NCT04671433)^5,9,11^.

The inexorable vision impairment associated with IRD may profoundly impact a patient’s quality-of-life (QoL), leading to higher rates of depression and anxiety^12,13^. Studies have shown that vision-related anxiety correlates with disability in this population^14^. X-linked RP imposes a particularly heavy humanistic, economic and emotional burden due to its early onset and rapid progression to blindness^12^. Patient-reported outcome (PRO) measures play a crucial role in assessing treatment efficacy and understanding its association with patient-perceived treatment benefits in a standardized manner^3,15,16^. These clinical outcome assessments convey the patient’s viewpoint on the disease’s impact on their life, and which aspects of visual dysfunction affect their emotional well-being^15,16^.

The Michigan Retinal Degeneration Questionnaire (MRDQ) and the Michigan Vision-related Anxiety Questionnaire (MVAQ) are two psychometrically validated PRO measures specifically developed for IRD patients, which were translated and linguistically validated for usage in Portuguese-speaking countries^17^. The MRDQ captures subjective disability across several domains representative of physiological visual function pathways^3,15^. Meanwhile, the MVAQ provides insight into vision-related anxiety, assessing the contributions of both cone and rod systems separately^16^.

Patients’ QoL is one of the seven priorities of the 2021–2025 National Eye Institute (NEI) Strategic Plan^18,19^. The authors underline the importance of incorporating patient perspectives in clinical research studies using vision-related QoL assessments based on PRO measures^18,19^.

To our knowledge, neither vision-related disability and anxiety nor any potential differences between males and females with *RPGR*-associated retinal degeneration have been explored before. The purpose of this study was to evaluate self-reported visual function and vision-related anxiety in a Portuguese cohort of male and female patients with *RPGR*-associated retinal degeneration using two validated PRO measures.

## Materials and methods

### Study design and participants

Cross-sectional study conducted at an IRD referral center in Portugal. Male and female patients with genetically confirmed *RPGR*-associated retinal degeneration were identified using the IRD-PT registry (retina.com.pt)^20^.

### Ethical statement

All patients provided written informed consent. The study was approved by the ethics committee of Coimbra University Hospital (protocol no GER/001/2016) and followed the tenets of the Declaration of Helsinki for biomedical research.

### Measures

We collected clinical data such as demographics (age and sex) and genetic testing results. Genetic variants were classified according to the guidelines of the American College of Medical Genetics and Genomics (ACMG).

The Early Treatment of Diabetic Retinopathy Study (ETDRS) introduced the ETDRS chart as a standardized visual acuity testing chart. Best corrected visual acuity (BCVA) was recorded as ETDRS letter score in the better-seeing eye. We used BCVA in the better-seeing eye as a surrogate for central visual function since PRO measures typically reflect binocular vision and in cases where there is a difference between eyes, visual function is primarily determined by the eye with better sight.

**Figure 1:**
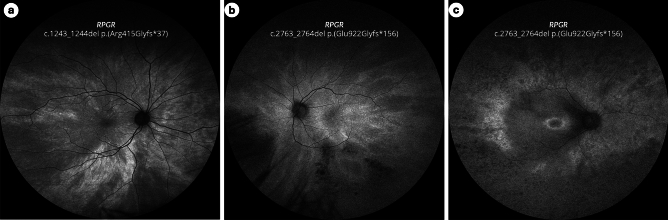
Retinal phenotype classification based on UW-FAF. (**a**) Radial pattern, (**b**) focal pattern, (**c**) male pattern.

Ultrawidefield fundus autofluorescence (UW-FAF) was used to classify retinal phenotype (Fig. 1): normal pattern, radial pattern (radial spoke–shaped reflexes extending from the central macular area in a radial pattern), focal pattern (focal pigmentary retinopathy patchy pigmentation with a radial reflex pattern), and male pattern^4^. We chose to include only the FAF phenotype of the better-seeing eye in our analysis based on the reasoning described above and the fact that a significant inter-eye asymmetry is not infrequent in heterozygous females^15^.

The MRDQ and MVAQ were developed using item response theory, factor analysis, and graded response models^3,13^. MRDQ measures the impact of visual impairment in daily tasks, containing 59 Likert-scaled question items across seven different dimensions: central vision, color vision, contrast sensitivity, scotopic function, photopic peripheral vision, mesopic peripheral vision, and photosensitivity^3,15^. On the other hand, MVAQ is a 14-item instrument with two domains: rod-function related anxiety, which included items like worrying about bumping into people/objects or walking on uneven ground at night, and cone-function related anxiety, with items such as worrying when reading or distinguishing colors^13,16^. Item response theory analysis results in a theta score, which represents the functional ability or anxiety of a person in the measured domain^3^. Theta scores are mean-centered at zero and follow a normal distribution with variance of 1^3^. They range between − 3 and + 3, where an increasing score indicates a higher severity in disability (MRDQ) or higher levels of anxiety (MVAQ)^21^. All participants completed the translated and linguistically validated Portuguese version of the Michigan Retinal Degeneration Questionnaire (MRDQ) and Michigan Vision-related Anxiety Questionnaire (MVAQ), where questions were read aloud by the interviewer, according to predefined guidelines^17^.

### Statistical analysis

Data normality was visually assessed and analyzed with the Shapiro–Wilk test. Normal distributed data was presented as mean and standard deviation (SD), and non-normal distributed data was presented as median and interquartile range (IQR). Using Kruskal–Wallis test, we searched for differences in age, BCVA of the better-seeing eye and all MRDQ and MVAQ theta scores between three groups based on FAF phenotype: males (M), females with male phenotype (FM) and females with radial or focal pattern (FRF). When a significant difference was detected, pairwise comparison was performed. Additionally, Spearman’s rank correlation was used to quantify the association between MVAQ theta scores and other variables such as age, BCVA of the better-seeing eye and MRDQ domains. Adjusted *p*-values according to Bonferroni correction for multiple comparison testing are presented. *P*-values were deemed significant if less than 0.05. All statistical analyses were performed using R 4.2.1 software (R Foundation for Statistical Computing).

## Results

Thirty-two patients (50% female) from 13 families with *RPGR*-associated retinal degeneration were included. The cohort’s clinical and demographic characteristics are shown in Table 1.

**Table 1:** Clinical and demographic characteristics.

Characteristic	Male	Female	Full sample
Age in years, mean (SD)	36.56 (13.70)	44.688 (15.24)	40.62 (14.84)
BCVA of better-seeing eye in ETDRS letters, median (IQR)	67 (60.75, 72)	83 (73.25, 85)	72 (65, 84.25)
FAF phenotype of better-seeing eye, n (%)			
Radial pattern	–	7 (43.8%)	7 (21.9%)
Focal pattern	–	4 (25.0%)	4 (12.5%)
Male pattern	16 (100%)	5 (31.2%)	21 (65.6%)

*BCVA* best corrected visual acuity, *ETDRS* early treatment of diabetic retinopathy study, *FAF* fundus autofluorescence, *IQR* interquartile range, *SD* standard deviation, *VUS* variant of uncertain significance.

Genetic analysis yielded 12 distinct variants in the *RPGR* gene, 8 of which were located in the ORF15 region. The genetic landscape of our cohort is further described in the Supplementary Table S1.

As expected, BCVA of the better-seeing eye was significantly higher in the FRF group compared to males and females with a male phenotype. There was no difference in BCVA between M and FM (Table 2).

**Table 2:** Comparison of clinical features and MRDQ and MVAQ scores between males, females with a male phenotype and females with a radial or focal pattern.

Variable	Males (M)	Females with male phenotype (FM)	Females with radial/focal pattern (FRF)	Overall *p*-value	Pairwise comparison *p*-value		
					M versus FM	FM versus FRF	M versus FRF
Age in years, median (IQR)	35.50 (26.00, 47.25)	59.00 (56.00, 62.00)	44.00 (31.00, 47.50)	0.115	–	–	–
BCVA of better-seeing eye in ETDRS letters, median (IQR)	67.00 (60.75, 72.00)	71.00 (70.00, 74.00)	85.00 (83.00, 85.00)	0.002*	0.4316	0.009*	0.003*
MRDQ theta scores, median (IQR)							
Central vision	0.312 (− 0.007, 0.995)	0.463 (− 0.343, 0.770)	− 0.894 (− 1.534, − 0.664)	0.004*	0.933	0.012*	0.008*
Color vision	0.414 (− 0.097, 0.972)	− 0.026 (− 0.699, 0.640)	− 1.095 (− 1.348, − 0.842)	0.001*	0.295	0.035*	0.002*
Contrast sensitivity	0.734 (0.358, 1.367)	0.519 (− 0.237, 0.836)	− 1.401 (− 1.401, − 0.862)	0.0001*	0.349	0.003*	0.0003*
Scotopic function	0.859 (0.401, 1.133)	− 0.007 (− 0.225, 0.592)	− 1.172 (− 1.505, − 0.935)	< 0.0001*	0.053	0.004*	0.0001*
Photopic peripheral function	0.798 (0.211, 1.399)	0.274 (− 0.060, 0.412)	− 1.262 (− 1.262, − 1.262)	< 0.0001*	0.138	0.016*	< 0.0001*
Mesopic peripheral function	0.999 (0.259, 1.239)	0.014 (− 0.147, 0.496)	− 1.771 (− 1.771, − 1.159)	< 0.0001*	0.032*	0.004*	< 0.0001*
Photosensitivity	0.048 (− 1.036, 0.520)	− 0.163 (− 0.634, − 0.084)	− 0.778 (− 1.093, − 0.527)	0.215	–	–	–
MVAQ theta scores, median (IQR)							
Rod-function anxiety	0.442 (− 0.003, 1.630)	0.180 (0.099, 0.881)	− 1.197 (− 1.197, − 0.415)	0.003*	0.835	0.014*	0.006*
Cone-function anxiety	0.675 (0.207, 0.915)	0.442 (0.359, 0.689)	− 0.563 (− 0.861, − 0.243)	0.017*	0.780	0.022*	0.022*

*BCVA* best corrected visual acuity, *ETDRS* early treatment of diabetic retinopathy study, *IQR* interquartile range, *MRDQ* Michigan retinal degeneration questionnaire, *MVAQ* Michigan vision-related anxiety questionnaire.

Asterisk (*) indicate a significant value (adjusted *p*-value < 0.05).

**Figure 2:**
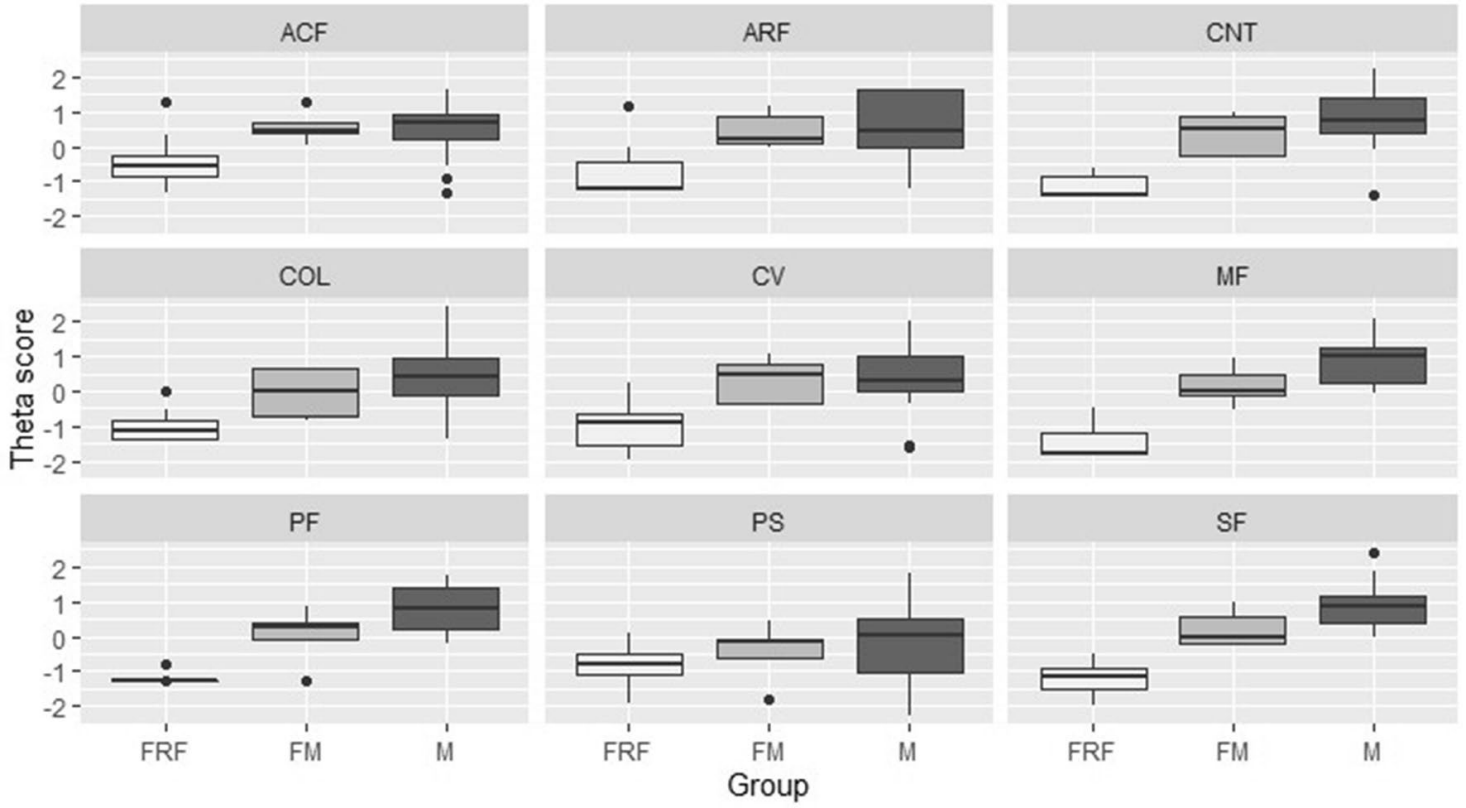
Comparison of MRDQ and MVAQ scores between males, females with a male phenotype and females with a radial or focal pattern. Note: ACF = cone-function related anxiety, ARF = rod-function related anxiety, CNT = contrast sensitivity, COL = color vision, CV = central vision, FM = females with male phenotype, FRF = females with radial or focal pattern, M = males, MF = mesopic peripheral function, PF = photopic peripheral function, PS = photosensitivity, SF = scotopic function.

Males and females with male pattern presented higher MRDQ theta scores than the FRF group in central vision, color vision, contrast sensitivity, scotopic function, photopic and mesopic peripheral function domains (Table 2). There was no significant difference in theta scores between males and FM, except for the mesopic peripheral function domain, in which males revealed greater disability (median 0.999) than FM (median 0.014). Similarly, median MVAQ scores for rod- and cone-function related anxiety were equally higher in males and FM compared to FRF (Table 2). The distribution of MRDQ and MVAQ domain scores across the three groups are shown in Fig. 2.

**Figure 3:**
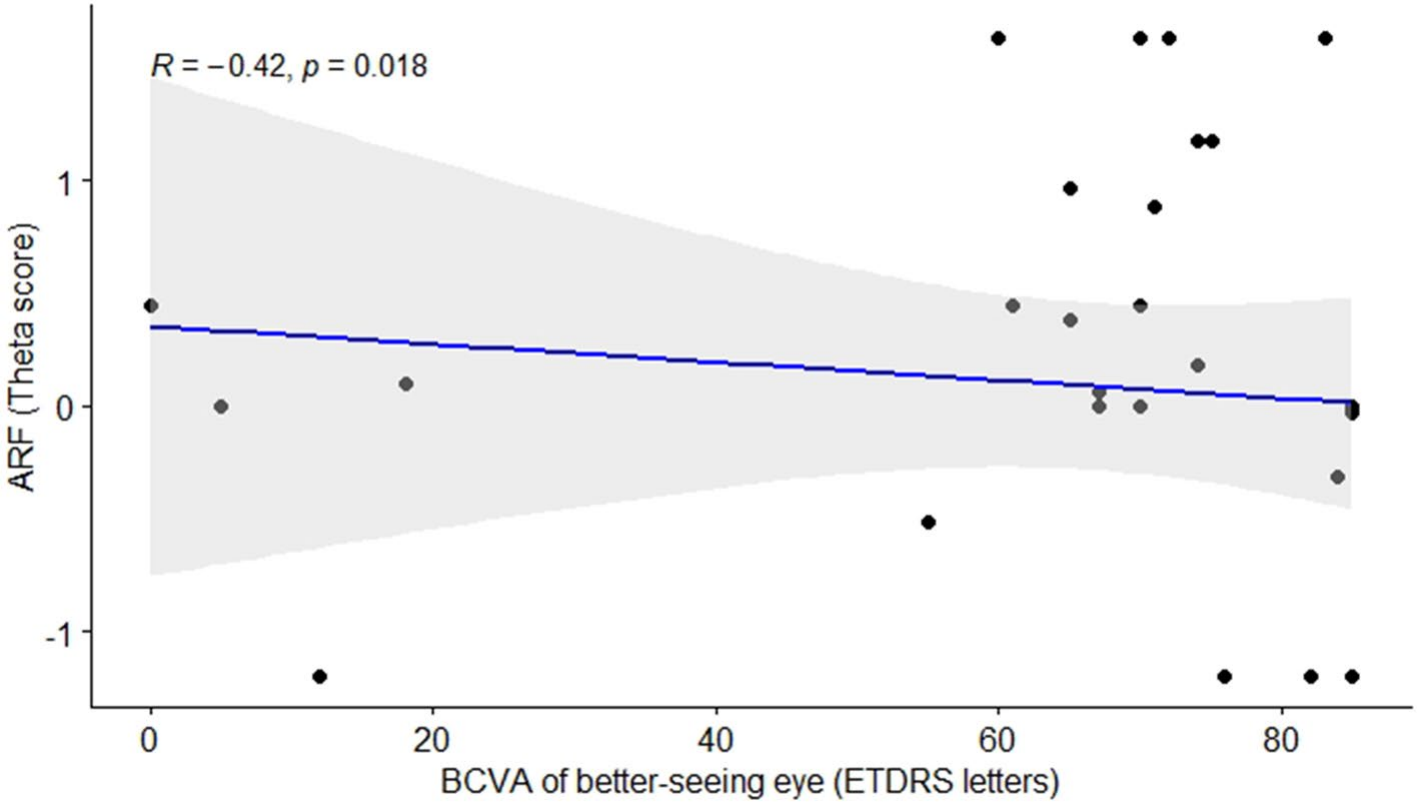
Negative correlation between MVAQ rod-function anxiety and BCVA. Note: ARF = rod-function related anxiety, BCVA = best corrected visual acuity.

**Figure 4:**
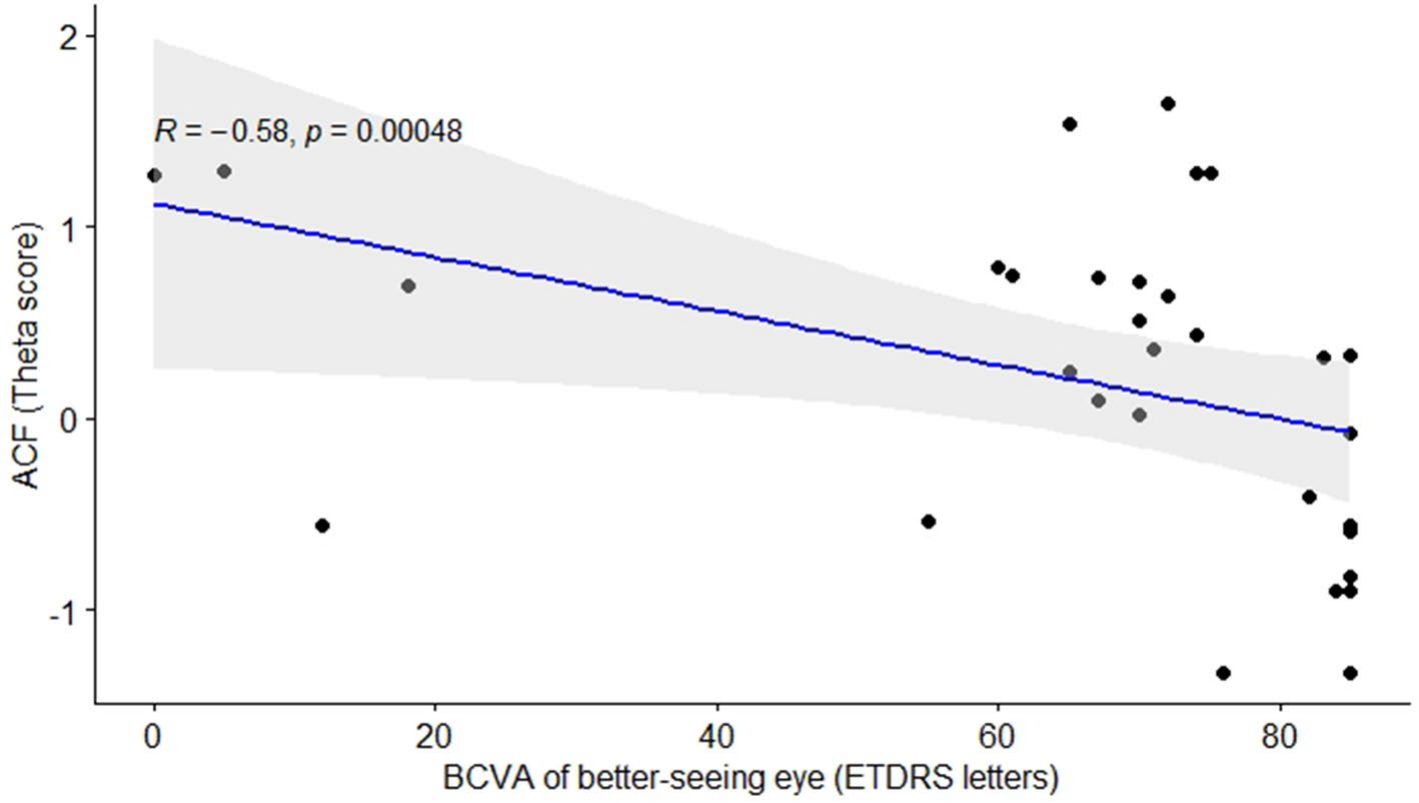
Negative correlation between MVAQ cone-function anxiety and BCVA. Note: ACF = cone-function related anxiety, BCVA = best corrected visual acuity.

Regarding BCVA of the better-seeing eye, the higher the visual acuity, the lower the cone and rod anxiety thetas (Table 3). These negative correlations between BCVA and both rod- and cone-function anxiety scores are graphically depicted in Figs. 3 and 4, respectively.

**Table 3:** Spearman’s rank correlation coefficient analysis between MVAQ scores and covariates.

Variable	Correlation with rod-function anxiety		Correlation with cone-function anxiety	
	Correlation coefficient, r	*p*-value	Correlation coefficient, r	*p*-value
Age	0.114	0.534	0.378	0.093
BCVA	− 0.417	0.02*	− 0.581	0.0005*
MRDQ domains				
Central vision	0.511	0.004*	0.773	< 0.0001*
Color vision	0.570	0.001*	0.699	< 0.0001*
Contrast sensitivity	0.635	0.0002*	0.782	< 0.0001*
Scotopic function	0.684	< 0.0001*	0.648	0.0001*
Photopic peripheral function	0.665	< 0.0001*	0.710	< 0.0001*
Mesopic peripheral function	0.699	< 0.0001*	0.655	< 0.0001*
Photosensitivity	0.524	0.003*	0.651	< 0.0001*
MVAQ domains				
Rod-function anxiety	1.00	–	0.786	< 0.0001*
Cone-function anxiety	0.786	< 0.0001*	1.00	–

*BCVA* best corrected visual acuity, *MRDQ* Michigan retinal degeneration questionnaire, *MVAQ* Michigan vision-related anxiety questionnaire.

Asterisk (*) indicate a significant value (adjusted *p*-value < 0.05).

Significant positive correlations between age and MRDQ scores related to central vision (r = 0.866; *p* < 0.0001), color vision (r = 0.585; *p* = 0.02), contrast sensitivity (r = 0.812; *p* = 0.0002), scotopic function (r = 0.769; *p* = 0.0008), photopic (r = 0.715; *p* = 0.003) and mesopic peripheral function (r = 0.737; *p* = 0.002) were found only in the male group. No significant correlations with age were observed in the FM or FRF groups.

Overall, positive correlations were found between every MRDQ domain and both rod- and cone-function related anxiety (Table 3). MRDQ mesopic peripheral function (r = 0.699; *p* < 0.0001) and scotopic function (r = 0.684; *p* < 0.0001) thetas had the strongest correlation with rod anxiety theta. MRDQ contrast sensitivity (r = 0.782; *p* < 0.0001) and central vision (r = 0.773; *p* < 0.0001) thetas had the strongest correlation with cone anxiety theta. Moreover, cone anxiety scores are positively correlated with concomitant rod anxiety scores and vice-versa (Table 3).

## Discussion

By evaluating self-perceived vision-related disability and anxiety in both male and female patients, this study makes a significant and innovative contribution to the current knowledge and adds a new dimension to our understanding of *RPGR*-associated retinal degeneration.

We highlight the importance of PRO measures in capturing the effect of visual loss on the patient’s function, mental health and QoL. In our cohort, both males and females with a male phenotype, presented increased disability scores in domains such as central vision, color vision, contrast sensitivity, scotopic function, photopic peripheral function and mesopic peripheral function. Both rod and cone related functional domains were affected in our population, which is explained by the rapid progression of XLRP leading to secondary cone degeneration.

In the male group, we found the highest perceived disability in the domains of scotopic function and mesopic peripheral function. This result is in line with data previously published by Karuntu et al. while evaluating visual function parameters in patients with RP using MRDQ^3^. Additionally, similar findings were described by Marques et al. in a cohort of *EYS*-associated retinal degeneration, where self-reported visual function and psychosocial impact of visual loss were evaluated using MRDQ and MVAQ^21^. On the other hand, in our group of females with male phenotype, highest perceived disability was observed in the domains of contrast sensitivity and central vision. Interestingly, the cohort of patients studied by Karuntu et al. also presented high scores of disability in contrast sensitivity and could be present at all stages of RP^3^. The lowest perceived disability score in our cohort of males and females with male phenotype was photosensitivity. This is consistent with the findings of Karuntu et al., whereas in the *EYS* cohort of Marques et al. color vision yielded the lowest disability score^3,21^. In the male group, there was a positive correlation between age and several domains of visual disability. This finding is in line with the natural history of the disease, where older men present more advanced stages of the disease, corresponding to increased levels of visual disability^1^. Additionally, albeit nonsignificant, there seems to be a difference in age between males and females with a male phenotype. We hypothesize that this age difference in our cohort may be attributed to several factors, such as our small sample size, a later onset of symptoms in females, and a lower awareness of the disease in female patients, leading to a delayed diagnosis.

Moreover, we observed that rod- and cone-function related anxiety scores were greater in males and females with male pattern, which is consistent with the self-perceived visual disability revealed by the MRDQ analysis. This hypothesis is supported by significant positive correlations found between anxiety scores and every MRDQ domain and BCVA. To date, very few studies have delved into the patient burden and psychosocial aspect of XLRP. In a review study, Chivers et al. described that people with RP reported a heavy psychosocial burden, having difficulty undertaking activities of daily living and maintaining independence^12^. Furthermore, they inferred from RP studies that XLRP ought to be associated with a greater burden than other forms of RP, with a greater impact on patients’ mental health due to the younger age of those affected and more rapid progression to advanced disease^12^. The significant correlations found in our study between self-perceived visual disability and vision-related anxiety are consistent with previous studies with IRD patients by Jayasundera et al. and Popova et al.^13,14^. Furthermore, our findings suggesting a stronger correlation of scotopic function with rod-function anxiety, and central vision with cone-function anxiety, correspond to the results presented by Popova et al. and highlight the interconnection of the phenomena measured by these two PRO questionnaires^13^.

By including female carriers of *RPGR* variants, this study underscores phenotype heterogeneity, showing that females with a male phenotype may experience similar levels of self-perceived disability and vision-related anxiety as males. It has been well documented that female carriers with a severe phenotype often face significant visual impairment early in life^4,5^. Including female carriers of *RPGR* variants in future studies is crucial for enhancing disease understanding and developing targeted therapies for both men and women^1,4,5^. The inclusion of standardized PRO measures in clinical trials and studies is essential for better representing the impact of visual loss on patients’ function and mental health, thereby providing adequate support^1,3^.

One limitation of this study is its cross-sectional nature, which prevents us from inferring about disease progression. Secondly, the sample size is relatively small as expected in a rare disease, and it is unequally distributed between the three groups analysed. Consequently, the statistical power to draw significant conclusions and detect any differences between groups is inevitably hindered. Thirdly, we acknowledge that using visual acuity as a surrogate of central visual function and biomarker of disease severity has its limitations and does not fully encompass all functional dimensions of vision. Lastly, our results may be influenced by ascertainment bias, as the cohort was drawn from an IRD referral center, where patients with more severe symptoms are more likely to be referred.

In conclusion, this is the first study to explore self-reported disability and vision-related anxiety using validated PRO measures in both males and females with *RPGR*-associated retinal degeneration. Our findings highlight the significant visual and psychological impairments experienced by female carriers of *RPGR* variants and underscore the need for a holistic approach to patient care. The MRDQ results can guide personalized low vision rehabilitation, while identifying patients with higher anxiety levels using the MVAQ can direct psychological interventions. This study demonstrates that males and females with a male phenotype show similar levels of increased vision-related anxiety and self-perceived visual disability, with rod- and cone-function related anxiety correlating positively with MRDQ domains and BCVA. Utilizing PRO measures like MRDQ and MVAQ enhances our understanding and supports more targeted, patient-centered therapeutic interventions.

### Supplementary Information

Supplementary Table S1.

## Data Availability

The datasets generated and analyzed during the current study are not publicly available due to the sensitive nature of the research supporting data but are available from the corresponding author on reasonable request.
