# Supplementary material for: Exploring self-reported visual function and vision-related anxiety in patients with RPGR-associated retinal degeneration
Source: Sci Rep. 2024 Jul 2;14:15189. doi: 10.1038/s41598-024-66170-2 (PMC11220147; doi:10.1038/s41598-024-66170-2)
Supplement: Supplementary file 1 — Supplementary Table S1. [file 41598_2024_66170_MOESM1_ESM.pdf]

Exploring self-reported visual function and vision-related anxiety in patients with *RPGR*-associated retinal degeneration

Nuno Gouveia[1,2,3], Oluji Chukwunalu[4], Carolina Oliveira[5], C. Henrique Alves[3,4,6,7], Rufino Silva[1,2,3], Joaquim Murta[1,2,3], João Pedro Marques*[1,2,3]

[1] Ophthalmology Department, Coimbra University Hospital, Coimbra, Portugal

[2] Faculty of Medicine, University of Coimbra (FMUC), Portugal

[3] Clinical Academic Center of Coimbra (CACC), Portugal

[4] Coimbra Institute for Clinical and Biomedical Research (iCBR), Faculty of Medicine, University of Coimbra (FMUC), Portugal

[5] Faculty of Psychology and Education Sciences, University of Coimbra (FPCEUC), Portugal

[6] Center for Innovative Biomedicine and Biotechnology (CIBB), University of Coimbra, Portugal

[7] Association for Innovation and Biomedical Research on Light and Image (AIBILI), Portugal

Supplementary Table S1

Genetic variant	Location	ACMG classification	Number of families	Number of patients
c.1243_1244del p.(Arg415Glyfs*37)	Exon 10	Pathogenic	2	5
c.2615_2616delAG p.(Glu872Glyfs*206)	ORF15	Likely pathogenic	1	5
c.2763_2764del p.(Glu922Glyfs*156)	ORF15	Pathogenic	1	5
c.2872del p.(Glu958Lysfs*131)	ORF15	Likely pathogenic	1	5
c.1261dup p.(Ser421Phefs*32)	Exon 11	Likely pathogenic	1	2
c.2501del p.(Glu834Glyfs*255)	ORF15	Pathogenic	1	2
c.3040del p.(Glu1014Argfs*75)	ORF15	Pathogenic	1	2
c.778+5G>A p.?	Exon 7	VUS	1	2
c.2064delG p.(Arg688Serfs*9)	ORF15	VUS	1	1
c.2379del p.(Gly794Glufs*21)	ORF15	VUS	1	1
c.2997_2998del p.(Glu1000Glyfs*78)	ORF15	Pathogenic	1	1
c.169T>G p.(Tyr57Asp)	Exon 3	VUS	1	1

Supplementary Table S1: Genetic landscape of the cohort. Note: ACMG = American College of Medical Genetics and Genomics, VUS = variant of uncertain significance.
